# Prevalence of SARS-CoV-2 IgG antibodies among dental teams in Germany

**DOI:** 10.1007/s00784-021-04363-z

**Published:** 2022-01-11

**Authors:** Maria Mksoud, Till Ittermann, Birte Holtfreter, Andreas Söhnel, Carmen Söhnel, Alexander Welk, Lena Ulm, Karsten Becker, Nils-Olaf Hübner, Andrea Rau, Stefan Kindler, Thomas Kocher

**Affiliations:** 1 Department of Oral and Maxillofacial Surgery/Plastic Surgery, University Medicine Greifswald, Walther-Rathenau-Str. 42a, 17475 Greifswald, Germany; 2 Institute for Community Medicine, University Medicine Greifswald, Greifswald, Germany; 3 Department of Restorative Dentistry, Periodontology Endodontology and Preventive and Pediatric Dentistry, University Medicine Greifswald, Greifswald, Germany; 4 Department of Prosthodontics, Gerodontology and Biomaterials, University Medicine Greifswald, Greifswald, Germany; 5 Friedrich Loeffler-Institute of Medical Microbiology, University Medicine Greifswald, Greifswald, Germany; 6 Central Unit for Infection Prevention and Control, University Medicine Greifswald, Greifswald, Germany

**Keywords:** Coronavirus, Epidemiology, Infection control, Occupational dentistry, Risk factor

## Abstract

**Objectives:**

During the corona pandemic, dental practices temporarily closed their doors to patients except for emergency treatments. Due to the daily occupational exposure, the risk of SARS-CoV-2 transmission among dentists and their team is presumed to be higher than that in the general population. This study examined this issue among dental teams across Germany.

**Materials and methods:**

In total, 2784 participants provided usable questionnaires and dry blood samples. Dry blood samples were used to detect IgG antibodies against SARS-CoV-2. The questionnaires were analyzed to investigate demographic data and working conditions during the pandemic. Multivariable logistic mixed-effects models were applied.

**Results:**

We observed 146 participants with positive SARS-CoV-2 IgG antibodies (5.2%) and 30 subjects with a borderline finding (1.1%). Seventy-four out of the 146 participants with SARS-CoV-2 IgG antibodies did not report a positive SARS-CoV-2 PCR test (50.7%), while 27 participants without SARS-CoV-2 IgG antibodies reported a positive SARS-CoV-2 PCR test (1.1%). Combining the laboratory and self-reported information, the number of participants with a SARS-CoV-2 infection was 179 (6.5%). Though after adjustment for region, mixed-effects models indicated associations of use of rubber dams (OR 1.65; 95% CI: 1.01–2.72) and the number of protective measures (OR 1.16; 95% CI: 1.01–1.34) with increased risk for positive SARS-CoV-2 status, none of those variables was significantly associated with a SARS-CoV-2 status in fully adjusted models.

**Conclusions:**

The risk of SARS-CoV-2 transmission was not higher among the dental team compared to the general population.

Clinical relevance.

Following hygienic regulations and infection control measures ensures the safety of the dental team and their patients.

**Supplementary Information:**

The online version contains supplementary material available at 10.1007/s00784-021-04363-z.

## Introduction

The coronavirus disease has affected nearly every aspect of our daily life and brought even the most robust health care systems to their knees [1, 2]. The high contagiousness and rapid global spread of SARS-CoV-2 have led to an unprecedented lockdown and restricted access to health and dental care [3]. Up to this point, the COVID-19 pandemic has caused approximately 5.32 million deaths worldwide [4]. Therefore, it would only be rational to say the pandemic is far from over.

SARS-CoV-2 is mainly transmitted through droplets and aerosol particles [5]. While droplets are larger and fall quickly to the ground due to gravity, aerosols tend to linger in the air and thereby pose a high risk of virus transmission when inhaled from nearby individuals or transferred to their mucosal surfaces [6]. Virus-loaded droplets and aerosols are generated while speaking, coughing, sneezing, and simply breathing. Moreover, those particles are released as a result of various medical procedures such as tracheal intubation, bronchoscopy, and dental treatment [7]. Aerosols generated during dental procedures, such as osteotomies, drilling, prophylaxis, and ultrasonic scaling, became recently a focal point with utmost urgency for policy-makers due to the fear of coronavirus transmission [8, 9]. Living coronavirus has been detected in the saliva [10]; thus, dentists and their team were presumed to be highly susceptible due to their frequent occupational exposure to aerosol-generating dental procedures (AGDP) compared to the general population. The question whether the dental team may even be “super spreaders” for the SARS-CoV-2 to their patients [11, 12] was widely discussed and caused major uncertainty. This led regulators and health authorities worldwide to call for postponing elective procedures and provide emergency-only treatment hoping to restrict the spread of the virus [13, 14].

Previous studies have pointed out that AGDP can be a source for transmitting pathogens, but there is a controversy surrounding the origin of those pathogens and of the pathogen load in AGDP [15, 16]. In an experimental in vivo study, Meethil et al. concluded that the risk for transmission of SARS-CoV-2 and other respiratory pathogens from aerosolized saliva in dental procedures is moderately low [7]. This observation was corroborated by in vivo studies, which showed that aerosol production during talking, exertional breathing, or coughing was higher than that during intubation or respiratory procedures [17]. A national online survey in the USA reported low prevalence and infection rates among practicing dentists in June 2020 [18]. Due to methodological limitations, such as high costs and limited capacity, studies addressing this issue on a larger scale using biospecimen are still sparse [19]. Expanding our understanding of SARS-CoV-2 transmission routes and pathogen origins within the dental office are essential factors to ensure the safety of the dental team and their patients so that they could regain unrestricted access to oral health care.

Responding to these shortcomings, we decided to investigate this issue with a national multicenter study conducted among dental team members in five regions in Germany. We aimed to investigate the risk of infection among the dental team compared to the general population, and clarify the impact of protective measures in preventing SARS-CoV-2 infection.

## Materials and methods

We carried out a cross-sectional study of 2998 individuals who work at licensed private dental practices in Germany between January and April 2021. The study protocol was approved by the Ethical Committee of the University Medicine Greifswald (BB 081/20a 03.12.2020). All participants signed an informed consent.

In January 2021, 5 urban regions in Germany with a higher incidence rate of coronavirus disease, according to the Robert Koch Institute (RKI), were selected. Those regions are (Appendix Table 1) (1) Berlin, (2) Hamburg, (3) Dresden, (4) Stuttgart, and (5) Cologne/Düsseldorf (in the following just named Cologne). In total, 7300 invitations were sent out to participate in this study. Each dental practice was asked to name three designated participants including a dentist, a dental nurse, and a dental prophylaxis nurse. Overall, 3305 participants from 1390 dental practices (equaling 4170 subjects) agreed to participate in this study and gave their written informed consent (response rate 79%).

**Fig. 1:**
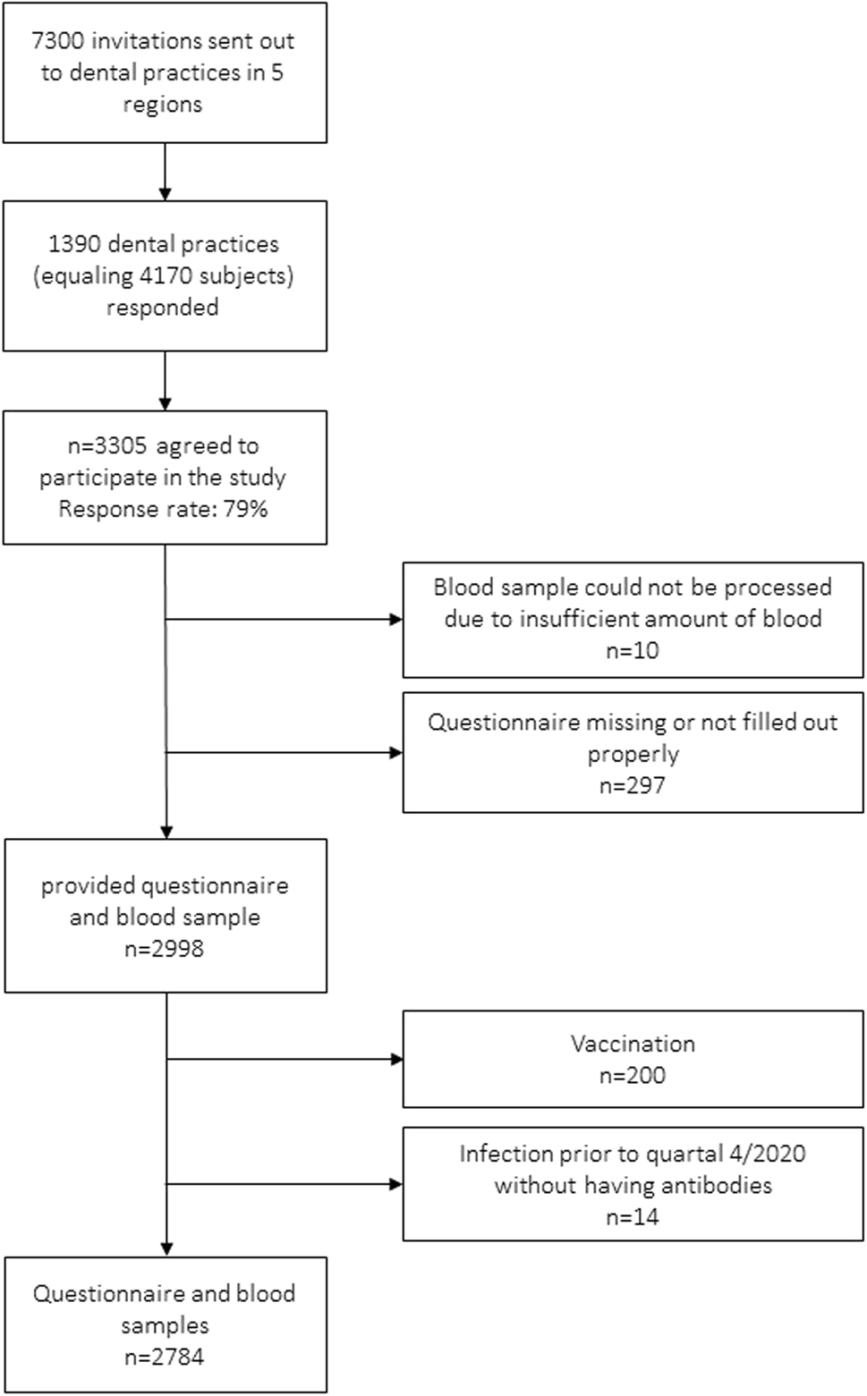
Flowchart of the study sample

Each participant received a study package that included a questionnaire and a dry blood collection set (EUROIMMUN Medizinische Labordiagnostika AG – Lübeck, Germany), both labeled with the same numerical identifier “ID.” IDs were automatically generated prior to sending out the study materials to ensure data privacy and enable the matching of self-reported data with biomaterials afterwards. Participants who did not complete the questionnaire (*n* = 297) or failed to provide a dry blood sample as instructed (*n* = 10) were excluded. By 21 April 2021, we received 2998 packages. A total of 200 participants had to be excluded due to vaccination. Furthermore, we excluded 14 participants who reported being previously tested positive for SARS-CoV-2 but no antibodies in their dry blood sample could be detected, thus leaving us with data from 2784 participants (Fig. 1).

Participants were asked if they had already suffered from a SARS-CoV-2 infection confirmed by a PCR test, had been vaccinated, or had treated patients positively tested for coronavirus disease. The remaining questions revealed how the practice activity and working hours were affected by the pandemic, the working circumstances in the practice, and implemented personal protective equipment (PPE). The survey encompassed the year 2020 and the answers were given in quarterly periods (Q1: January–March, Q2: April–June, Q3: July–September, Q4: October–December). For the present analyses, we focused on the answers from the second, third, and fourth quarters.

Participants collected a capillary blood sample from the fingertip which were tested for anti-SARS-CoV-2 IgG antibodies at the Friedrich Loeffler-Institute of Medical Microbiology (University Medicine Greifswald). Testing for antibodies against the S1-domain of the SARS-CoV-2 spike protein was performed in accordance with the manufacturer’s instructions (anti-SARS-CoV-2-ELISA; Euroimmun, Lübeck, Germany). The diagnostic sensitivity of this assay was reported to be 43.7% in samples taken until day 10 after symptom onset or positive direct detection and 94.4% in samples collected after day 10 and specificity was reported to be 99.6% [20]. A participant was considered as having had a SARS-CoV-2 infection if he/she had SARS-CoV-2 antibodies or reported having a positive SARS-CoV-2 PCR test before our study period. Data from 24 individuals with a borderline laboratory finding without reporting a positive SARS-CoV-2 PCR test were set to missing.

As for our control population, we intended to select the representative SOEP-CoV panel [21]. This study collected biospecimen (dry blood sample and a swab sample from the mouth and nose) from a nationwide population sample drawn from the German Socio-Economic Panel (SOEP).

### Statistical analysis

The prevalence of participants with positive SARS-CoV-2 IgG antibodies and SARS-CoV-2 infections was calculated as relative frequencies. Stratified by region and SARS-CoV-2 status, continuous data were expressed as mean and standard deviation, while categorical data were reported as relative frequencies. Associations of potential protective and risk factors for a SARS-CoV-2 infection were analyzed by a random intercept logistic mixed-effects model nested in the physician’s practice and adjusted for the region. Results were reported as odds ratio (OR), 95% confidence interval (CI), and *p* value. A *p* < 0.05 was considered statistically significant. All analyses were conducted with Stata/SE 16.1 (StataCorp, 2019, Stata Statistical Software: release 16, College Station, TX: StataCorp LLC).

## Results

**Fig. 2:**
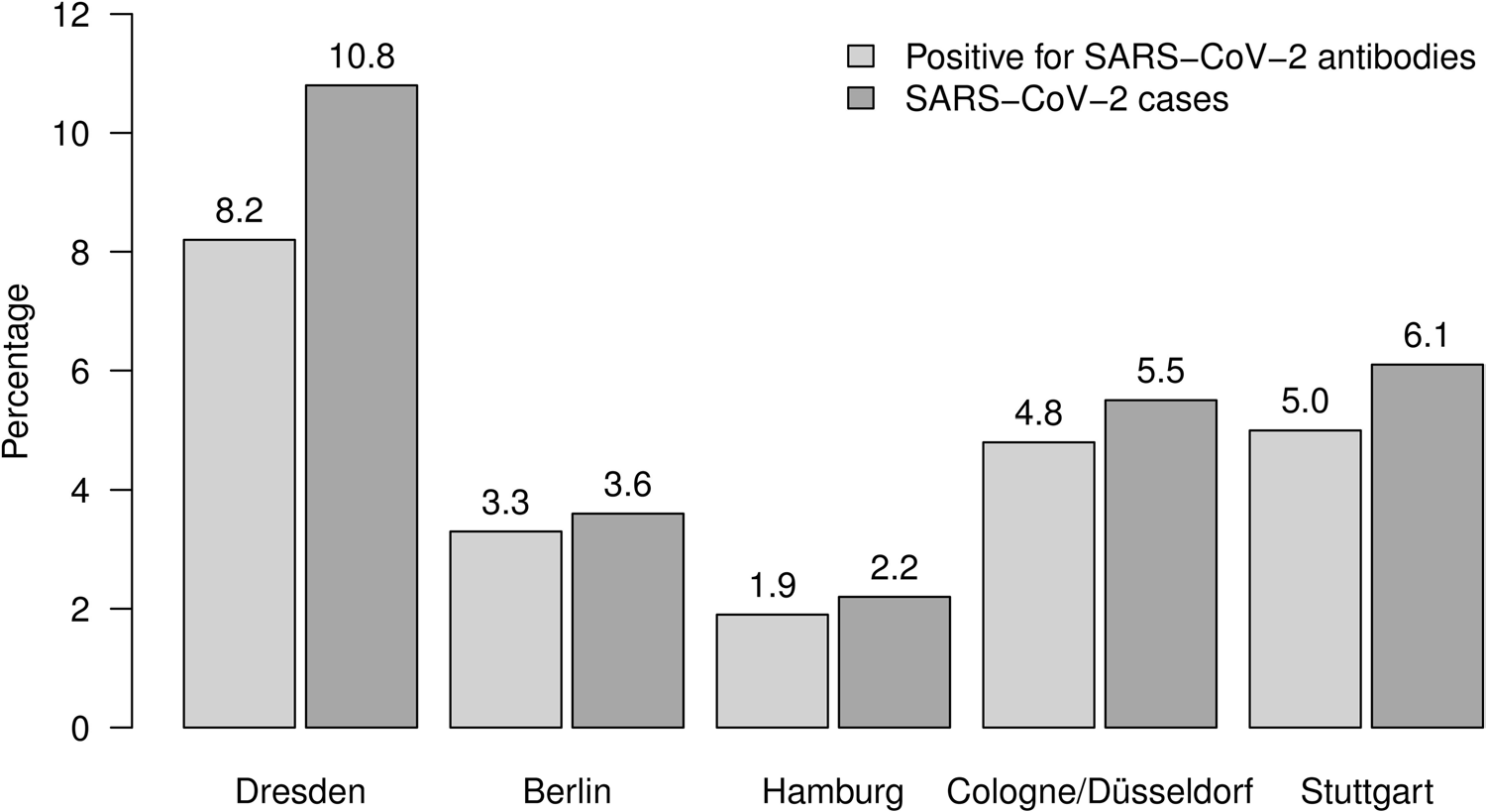
Percentage of participants positive for SARS-CoV-2 antibodies and percentage of SARS-CoV-2 cases stratified by region

We examined 2784 dental team members from 1125 offices in Germany (Table 1). We observed 146 participants with positive SARS-CoV-2 IgG antibodies (5.2%) and 30 subjects with a borderline finding (1.1%). In total, 74 out of the 146 participants with SARS-CoV-2 IgG antibodies did not report a positive SARS-CoV-2 PCR test (50.7%). On the other hand, 27 participants without SARS-CoV-2 IgG antibodies did report a positive SARS-CoV-2 PCR test (1.1%). When combining the laboratory and self-reported information, the number of participants with a SARS-CoV-2 infection was 179 (6.5%). The frequency of SARS-CoV-2 IgG antibodies was highest in Dresden, followed by Stuttgart and Cologne (Fig. 2, Table 1). In comparison to Hamburg, the risk for SARS-CoV-2 IgG antibodies was significantly higher in Dresden (OR = 6.11; 95% CI: 2.77–13.47; *p* < 0.001), Cologne (OR = 2.73; 95% CI: 1.15–6.48; *p* = 0.023), and Stuttgart (OR = 3.06; 95% CI: 1.21–7.76; *p* = 0.018) but not in Berlin (OR = 1.70; 95% CI: 0.72–4.02; *p* = 0.227).

**Table 1:** Characteristics of the study population stratified by location

Location	Total (*n* = 2784)	Dresden (*n* = 968)	Berlin (*n* = 696)	Hamburg (*n* = 365)	Cologne (*n* = 476)	Stuttgart (*n* = 279)
Number of offices	1125	379	284	155	193	114
Age in years	44.8 (12.5)	46.6 (11.6)	45.2 (12.5)	44.0 (13.4)	42.7 (12.4)	42.2 (13.3)
Women	84.0%	88.4%	84.1%	77.8%	82.8%	79.1%
Occupational group						
Dentist	34.9%	33.4%	35.5%	37.0%	34.9%	36.2%
Dental nurse	34.3%	34.7%	33.5%	33.4%	35.3%	34.8%
Dental prophylaxis nurse	30.8%	31.9%	31.0%	29.6%	29.8%	29.0%
Positive SARS-CoV-2-IgG antibody	5.2%	8.2%	3.3%	1.9%	4.8%	5.0%
Positive SARS-CoV-2 PCR test	3.8%	7.6%	2.0%	0.3%	2.1%	2.2%
Positive SARS-CoV-2 antibody or PCR test	6.5%	10.8%	3.6%	2.2%	5.5%	6.1%
Working time with patient in hours	29.5 (8.6)	29.5 (7.9)	28.7 (8.5)	29.8 (8.5)	30.6 (8.9)	29.3 (10.5)
Protective equipment						
FFP mask	74.2%	60.6%	79.6%	86.0%	80.5%	81.4%
Visor	63.9%	67.5%	66.0%	53.2%	63.2%	61.7%
Safety goggles	77.7%	67.3%	81.3%	87.7%	85.3%	78.5%
Bonnet	19.2%	16.4%	20.1%	17.8%	20.6%	26.2%
Single-use work coat	11.0%	9.6%	9.9%	11.5%	12.8%	14.7%
Rubber dam	12.6%	7.9%	10.2%	20.3%	14.5%	21.9%
Number of protective measures	2.6 (1.2)	2.3 (1.2)	2.7 (1.2)	2.8 (1.2)	2.8 (1.2)	2.8 (1.3)
Distancing measures, yes	95.7%	95.0%	95.5%	96.7%	96.6%	95.3%
Number of aerosol-generating devices	2.5 (1.0)	2.6 (0.9)	2.4 (0.9)	2.2 (1.2)	2.7 (1.0)	2.6 (1.1)
Ventilation systems, yes	27.4%	19.2%	31.1%	31.6%	30.3%	35.8%
Ventilation after each treatment, yes	86.8%	75.1%	90.6%	92.2%	96.2%	94.9%
Pre-treatment mouthwash, yes	75.8%	65.3%	80.0%	82.1%	83.1%	80.9%
Size of the practice rooms						
< 15 m^2^	39.3%	39.4%	34.0%	38.0%	45.7%	43.3%
15–20 m^2^	40.3%	41.6%	38.3%	43.4%	38.9%	39.5%
≥ 20 m^2^	20.4%	19.1%	27.7%	18.6%	15.5%	17.2%

Data are expressed as percentages for categorical data or as mean and standard deviation for continuous data

**Fig. 3:**
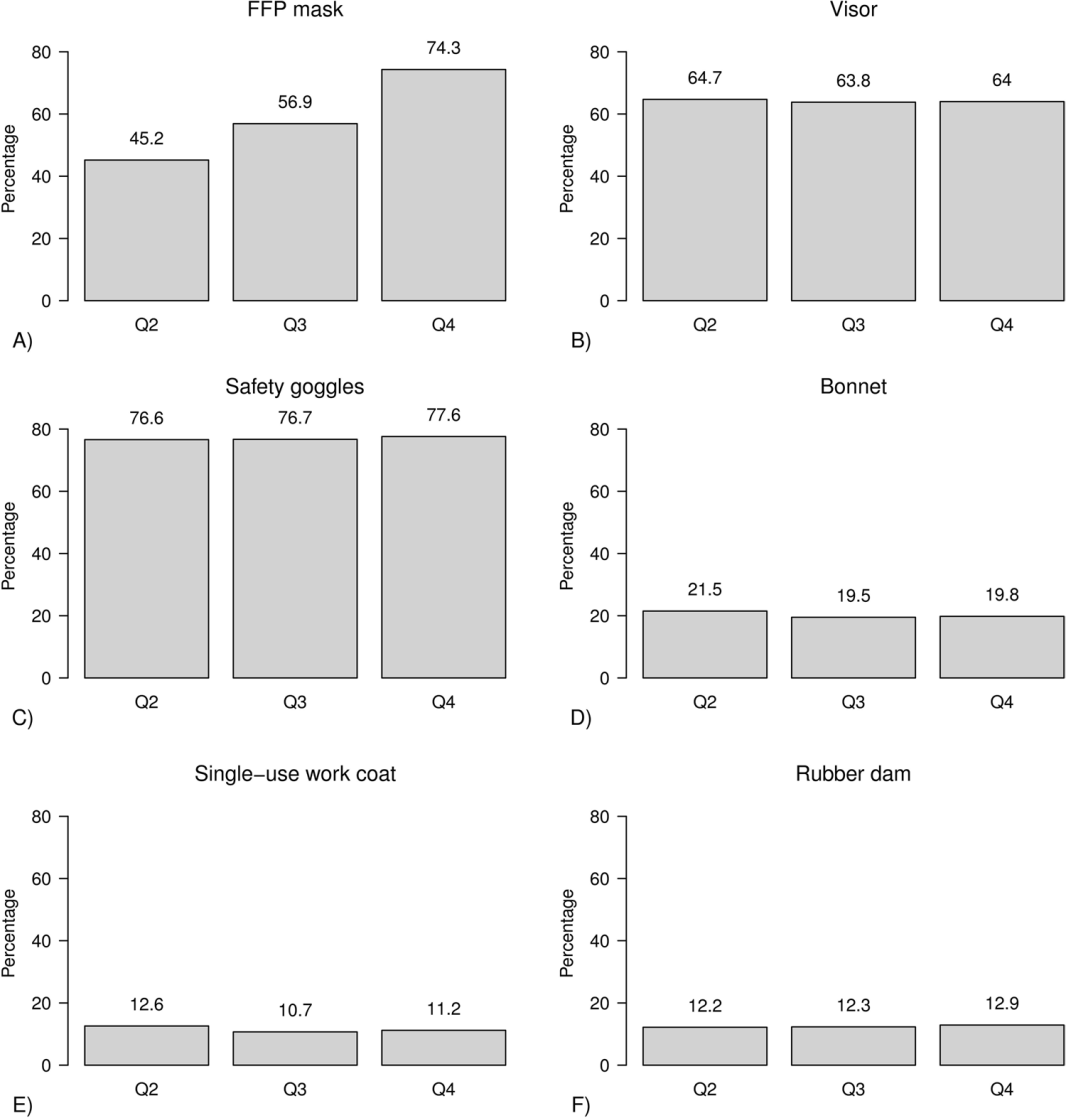
Percentage of study participants using different protective measures over the last three quarterly periods in 2020

Usage of filtering face pieces (FFP) masks increased from 48 to 75% from the 2^nd^ to the 4^th^ quarter of 2020, whereas visors and goggles were used regardless of the timeline in 60% and 80% of all participants, respectively. AGDP working time dropped about 3 h from the 28 h/week to 25 h/week from the 1^st^ to 2^nd^ quarter but then increased steadily up to the 4^th^ quarter to reach 29 h/week. The frequency of applying distancing measures was comparable (i.e., about 96%) in all regions (Fig. 3).

In Dresden, protective equipment (FFP masks about 20% less, safety googles about 10% less than average), and room ventilation after patient treatment (about 10% less), and ventilation systems (about 15% less), and pre-treatment mouthwash and ventilation after each treatment were less often conducted in Dresden than in other regions.

In logistic mixed-effects models adjusted for regions, the risk for a SARS-CoV-2 infection was significantly associated with using a rubber dam (OR = 1.65, 95% CI: 1.01–2.72) and with the number of protective measures (OR = 1.16, 95% CI: 1.01–1.34; Table 2). No such associations were observed for the other protective equipment or ventilation measures. Age, sex, occupational group, working time with the patient, application of distancing measures, number of aerosol-generating devices, use of ventilation systems, ventilation after each examination, pre-treatment mouthwash, and size of the practice rooms were not significantly associated with a SARS-CoV-2 infection. In a multivariable logistic mixed-effects model including age, sex, occupational group, working time with patient, use of FFP mask, use of visor, use of rubber dam, application of distancing measures, number of aerosol-generating devices, availability of ventilation systems, pre-treatment mouthwash, and size of practice rooms, none of those variables was significantly associated with a SARS-CoV-2 status. Particularly, reported significant association between using a rubber dam and SARS-CoV-2 status attenuated and turned non-significant (OR = 1.44, 95% CI 0.82–2.53; *p* = 0.206) (Table 2).

**Table 2:** Associations of potential protective or risk determinants with SARS-CoV-2 status

	SARS-CoV-2 negative	SARS-CoV-2 positive	Odds ratio (95% confidence interval)
Age in years	44.8 (12.4)	44.2 (13.1)	0.99 (0.98; 1.01)
Sex			
Men	16.2%	11.7%	0.78 (0.49; 1.25)
Women	83.8%	87.7%	Reference
Diverse	0.1%	0.6%	
Occupational group			
Dentist	35.2%	29.6%	Reference
Dental nurse	34.3%	36.3%	1.25 (0.84; 1.86)
Dental prophylaxis nurse	30.5%	34.0%	1.32 (0.88; 1.97)
Working time with patient in hours	29.6 (8.5)	28.4 (10.0)	0.98 (0.96; 1.00)
Protective equipment			
FFP mask	74.3%	72.1%	1.21 (0.81; 1.79)
Visor	63.3%	70.4%	1.30 (0.90; 1.88)
Safety goggles	77.9%	75.4%	1.13 (0.75; 1.69)
Bonnet	19.2%	20.1%	1.11 (0.72; 1.71)
Single-use work coat	10.9%	14.0%	1.43 (0.86; 2.39)
Rubber dam	12.4%	15.1%	1.65 (1.01; 2.72)*
Number of protective measures	2.6 (1.2)	2.7 (1.3)	1.16 (1.01; 1.34)*
Distancing measures, yes	95.7%	94.4%	0.75 (0.36; 1.62)
Number of aerosol-generating devices	2.5 (1.0)	2.6 (1.0)	1.01 (0.85; 1.22)
Ventilation systems, yes	27.8%	24.0%	0.90 (0.60; 1.36)
Ventilation after each treatment, yes	87.1%	84.4%	1.15 (0.70; 1.89)
Pre-treatment mouthwash, yes	75.8%	75.0%	1.22 (0.81; 1.83)
Size of the practice rooms			
< 15 m^2^	39.7%	34.3%	0.83 (0.53; 1.29)
15–20 m^2^	39.9%	45.6%	1.11 (0.72; 1.69)
≥ 20 m^2^	20.4%	20.1%	Reference

Data are expressed as percentages for categorical data or as mean and standard deviation for continuous data. Odds ratios were derived from random intercept mixed-effects models with logit link nested in the physician’s practice and adjusted for region. *p < 0.05

Unfortunately, the SOEP’s control subjects had already been recruited until mid-November 2020, before the onset of the 3^rd^ wave in Germany. Thus, we had to switch to other cohorts with much more constraints. In the general German population, the cumulative incidence of PCR-validated SARS-CoV-2 infections reported to the RKI for the time between October 1, 2020, and April 15, 2021, was 5.0% for Dresden, 4.1% for Berlin, 3.4% for Hamburg, 3.7% for Cologne, and 3.5% for Stuttgart. In the SeBluCo study, the RKI measured SARS-CoV-2 antibodies in about 5000 blood donors every 14 days in 28 regions of Germany. In that study, the prevalence of positive antibodies was about 3% in January, 6% in February, and 7% in March across Germany.

## Discussion

Our data allow a tentative conclusion that dentists and their teams are not at higher risk for patient-dentist transmission of SARS-CoV-2. With statistical nil results, it has to be kept in mind that our study may not have been large enough to find essential differences [22]. Our tentative conclusion is composed of our results as well as information from the reported literature. Probably, the widespread use of FFP masks, ventilation measures, and the low virus load in the AGDP contributed to these findings [23]. To our knowledge, this is the first study in Germany that evaluated SARS-CoV-2 antibodies among the dental team using dry blood assays.

During the first wave of SARS-CoV-2 in 2020, dentists worldwide suffered from a severe shortage of PPE [24, 25]. Moreover, the effectiveness of various types of PPE and ventilation procedures was not yet thoroughly investigated. The continuous increase use of PPEs, mouthwash, and ventilation procedures peaked or plateaued in the 4^th^ quarter of 2020 in Germany. We assume a similar pattern since we have no data on their use in the 1^st^ quarter 2021. Thus, when the 2^nd^ corona wave hit Germany in winter 2020, most dental teams were very well prepared because appropriate material was available in sufficient quantities and knowledge was widely disseminated on how to mitigate SARS-CoV-2 exposure. Our findings contrast a study with over 1500 dental team members, which was performed in May 2020 amidst the peak of the 1^st^ wave in the UK [19]. During this time, neither PPEs were available in sufficient quantities, nor was knowledge widespread on how to minimize exposure. The UK infection rate among dental team members was twice as high as in the general population (16.3% vs. 6–7%). Dental receptionists without direct patient contact were less often SARS-CoV-2 antibody-positive than dentists or dental nurses (6.3%, 16.9%, and 16.7%, respectively). In our cohort, though without attaining statistical significance, we observed a similar trend of increased infection rates across the different dental occupations: prophylaxis nurses, who usually work alone, had a higher rate (7.2%) than dental nurses or dentists (6.8% and 5.5%, respectively). Nurses working alone had probably a less efficient suction and may be exposed to a somewhat higher risk [7]. Higher seropositivity rates among the dental team in comparison to the general population were not confirmed in an Italian study which traced the serological status of the dental team members from June to September 2020 in the Lombardy region [26]. These two datasets should help the public to understand that dental practices with continuous use of PPE measures are safe, and that avoidance or delay of needed and urgent health care is not necessary [27].

Our results showed that the prevalence of SARS-CoV-2 was highest in the region of Dresden, where the lowest use of personal protective equipment, pre-treatment mouthwash, and ventilation systems was recorded. This observation goes hand in hand with the highest prevalence SARS-CoV-2 positivity in the German general population. Thus, we cannot tease out whether the higher SARS-CoV-2 prevalence in the general population or the lower use of PPE, mouthwash, or ventilation measures has led to the higher exposure with SARS-CoV-2. There are three possible explanations why the usage of preventive measures was lowest in Dresden: Firstly, Dresden was the only location in former East Germany included in our sample, and East German dentists still earn less than their West German counterparts. Therefore, they may be less willing to spend money on protective measures. Secondly, the attitude of dental teams in Dresden reflects the general population’s doubts about the transmission and health-related consequences of SARS-CoV-2 and regards the use of PPE as unnecessary. Thirdly, the first wave in spring 2020 in Germany did not hit the population in Dresden hard: thus, dentists extrapolated their experience from the 1^st^ wave to the 2^nd^ wave and deemed additional PPE measures unnecessary.

Our results showed no significant association between the regular ventilation after treatment and use of ventilation systems and SARS-CoV-2 infection. This might be due to the very high rate of regular ventilation (90.7 to 95.0%) in all offices. Furthermore, about 30% of the offices used air ventilation systems. The use of regular ventilation after treatment or the installation of air ventilation systems has been proven to be crucial to eliminate aerosol enrichment in dental operatories [28]. Besides, 90% of our participants used FFP masks, which have a higher filtration efficiency for small viral particles than standard surgical masks [23]. FFP masks are subcategorized based on their total filtration efficacy into FFP1 (at least 80%), FFP2 (at least 94%), and FFP3 (minimum of 99%) [29]. In particular, FFP2 masks with correct fitting have been proven to maintain their bacterial filtration over time and provide the ultimate protection in both directions, i.e., the dentist and the patient, which is critical during dental treatment when airborne pathogens are involved [23, 30]. This high proportion of FFP mask users probably contributed to the low percentage of SARS-CoV-2 antibody–positive participants among the dental team. Another topic in this context is aerosols generated during dental treatments. In the public discussion, it was assumed that the visible spray equates to an infectious aerosol, but both the public and dentists were not aware that the coolant dilutes the saliva up to 200-fold. Meethil et al. treated 28 asymptomatic COVID-19 patients with different dental procedures and a high-suction evacuator. In these patients, the viral load in the saliva varied between 10^3^ and 10^6^ copies/ml; however, no virus was detected in any aerosol deposited on either the dentist or assistant nurse [7]. Thus, the danger of the dental aerosol was probably overestimated in the public discussion. Our study was not able to figure out which measure mitigated the infection risk, but we can conclude that the sum of the measures was sufficient to limit the virus spread in the dental office.

Interestingly, the use of rubber dam was positively associated with a SARS-CoV-2 infection in a first sparsely adjusted model, which only considered the cluster structure of the data (i.e., clustering of practices within region). However, after comprehensive adjustment, use of rubber dam was non-significantly associated with a SARS-CoV-2 status (OR = 1.44, 95% CI 0.82–2.53; *p* = 0.206). Nevertheless, we would like to stress that a thoughtful handling of rubber dams is essential to avoid exposure to saliva, especially as many dentists are not using rubber dams regularly. National German guidelines strongly encouraged the use of rubber dam when possible, in addition to a high-power suction as a protective measure when treating patients during the pandemic. This was echoed by many researchers who used rubber dam prior to various dental treatments [31].

The selection of practices in our study was not representative because we had no complete directory of practicing dentists in the selected regions, which were a priori chosen because of a high infection incidence at the planning stage. However, the most significant limitation of our study is the lack of prevalence data of the general population at the exact location and time. Unfortunately, our planned control population was examined before the 2^nd^ wave started in Germany; thus, a comparison would have been meaningless. To answer the question whether continuous work with AGDP poses a higher risk to the dental team, we accessed public domain data based on either PCR or antibody tests. Our results demonstrated a slightly higher prevalence of antibodies than the number of PCR-confirmed cases in Germany reported by the RKI [32]. However, the number of PCR-confirmed cases underestimates the actual situation of SARS-CoV-2 infection, because PCR tests were mainly restricted to patients presenting moderate to severe symptoms [33] or putatively exposed subjects, but not to inconspicuous persons. From our results, we can deduce the ratio of underreporting: 74 out of 146 participants with SARS-CoV-2 antibodies did not report a positive PCR test (50.7%). Correcting the PCR values reported by the RKI with a presumed 50% underreporting leads to a comparable prevalence (10.0% for Dresden, 8.2% for Berlin, 6.8% for Hamburg, 7.4% for Cologne, and 7.0% for Stuttgart). Another study (SeBluCo) conducted by the RKI investigated SARS-CoV-2 antibodies in approximately 5000 blood donors every 14 days in 28 regions in Germany [34]. This study reported antibody prevalences of 3%, 6%, and 7% in January, February, and March, respectively. In summary, both studies conducted by the RKI estimated population prevalences between 3 and 10%. A retrospective US study evaluated the frequency of coronavirus disease among patients seeking dental treatment from June to December 2020 and reported a similar frequency among patients compared to the general population [35]. During the sample collection period, the Alpha became the dominant variant in the study region.

In light of those numbers, we believe that the prevalence of SARS-CoV-2 antibodies among the dental team is comparable to the general population in Germany. However, we have to underpin that these conclusions are based on weak evidence. Our results, in line with other internationally published studies, confirm that current measures to reduce transmission continue to work against further transmission of SARS-CoV-2.

## Supplementary Information

Below is the link to the electronic supplementary material.

Supplementary file1 (DOCX 16 KB)

## Data Availability

The datasets generated during and/or analyzed during the current study are not publicly available due to the data protection act. Deidentified individual participant data that underlie the results reported in this manuscript will be available to the scientific community on request. Applicants interested must provide a proposal form which entails the scientific aim of the usage of the data provided and the institutional review board approval of the research proposal. All proposals should be directed to the corresponding author.
