# Supplementary material for: Prevalence of SARS-CoV-2 IgG antibodies among dental teams in Germany
Source: Clin Oral Investig. 2022 Jan 11;26(5):3965–74. doi: 10.1007/s00784-021-04363-z (PMC8751466; doi:10.1007/s00784-021-04363-z)
Supplement: Supplementary file 1 — Supplementary file1 (DOCX 16 KB) [file 784_2021_4363_MOESM1_ESM.docx]

**Prevalence of SARS-CoV-2 IgG Antibodies among Dental Teams in Germany**

Maria Mksoud ^1^, Till Ittermann ^2^, Birte Holtfreter ^3^, Andreas Söhnel ^4^, Carmen Söhnel ^1^, Alexander Welk ^3^, Lena Ulm ^5^, Karsten Becker ^5^, Nils-Olaf Hübner ^6^, Andrea Rau ^1^, Stefan Kindler ^1*^, Thomas Kocher ^3*^

1 Department of Oral and Maxillofacial Surgery/Plastic Surgery, University Medicine Greifswald, Greifswald, Germany

2 Institute for Community Medicine, University Medicine Greifswald, Greifswald, Germany

3 Department of Restorative Dentistry, Periodontology Endodontology and Preventive and Pediatric Dentistry, University Medicine Greifswald, Greifswald, Germany

4 Department of Prosthodontics, Gerodontology and Biomaterials, University Medicine Greifswald, Greifswald, Germany

5 Friedrich Loeffler-Institute of Medical Microbiology, University Medicine Greifswald, Greifswald, Germany

6 Central Unit for Infection Prevention and Control, University Medicine Greifswald, Greifswald, Germany

* These authors contributed equally

**Corresponding author:**

Maria Mksoud

Department of Oral and Maxillofacial Surgery/Plastic Surgery

University Medicine Greifswald

Walther-Rathenau-Str. 42a

17475 Greifswald, Germany

E-mail: mksoudm@uni-greifswald.de

Tel.: +49 3834 86 7180

Fax.: +49 3834 86 7183

Appendix Table 1. Detailed overview of the districts and regions included in this study

| 1. State of Berlin |
| --- |
| 1. State of Hamburg |
| 1. Dresden (State of Saxony) |
| Dresden city |
| Sächsische Schweiz-Osterzgebirge district |
| Meißen district |
| Bautzen district |
| Erzgebirgskreis district |
| Mittelsachsen district |
| Chemnitz city |
| 1. Stuttgart (State of Baden-Württemberg) |
| Stuttgart city |
| Ludwigsburg district |
| Schwäbisch Hall district |
| Esslingen city |
| Göppingen city |
| 1. Cologne/Düsseldorf (State of North-Rhine Westphalia) |
| Düsseldorf city |
| Mettmann district |
| Hagen city |
| Cologne city |
| Oberbergischer district |
| Hamm city |
